# Comorbidities in Clinical and Polysomnographic Features of Obstructive Sleep Apnea: A Single Tertiary Care Center Experience

**DOI:** 10.1007/s44197-022-00067-z

**Published:** 2022-10-03

**Authors:** Hamdan Al-Jahdali, Anwar E. Ahmed, Al-Harbi Abdullah, Khan Ayaz, Almuttari Ahmed, ALGamedi Majed, Alyami Sami, Almuhayshir Amirah, Dahman Bassam

**Affiliations:** 1 King Saud Bin Abdulaziz University for Health Sciences, Ministry of the National Guard - Health Affairs, Riyadh, Saudi Arabia; 2 King Abdullah International Medical Research Center, Riyadh, Saudi Arabia; 3 Department of Preventive Medicine and Biostatistics, F. Edward Hébert School of Medicine, Uniformed Services University of the Health Sciences, Bethesda, MD USA; 4 Henry M Jackson Foundation for the Advancement of Military Medicine, Bethesda, MD USA; 5 Department of Health Behavior and Policy, Virginia Commonwealth University, Richmond, VA USA; 6 Pulmonary Division, King Saud University for Health Sciences, Medical Director of Sleep Disorders Center, King Abdulaziz Medical City, Riyadh, Saudi Arabia

**Keywords:** Obstructive sleep apnea, Comorbidities, Hypertension, Diabetes mellitus, Coronary artery disease, Congestive heart failure, Obesity, Saudi

## Abstract

**Background:**

Research on obstructive sleep apnea (OSA) is inadequate in Saudi Arabia, particularly among patients with comorbidities. This study investigates comorbidities in patients with different severity of apnea based on the Apnea–Hypopnea Index (AHI).

**Methods:**

The retrospective charts review that included a cohort of 4391 patients who underwent polysomnography (PSG) between 2003 and 2019. The AHI is classified into four ordinal groups: normal, mild, moderate, and severe. Ordinal logistic regression was used to model proportional odds of a higher AHI category.

**Results:**

Gender was distributed equally in the study sample. The average age was 49.6 ± 14.8 years and the average AHI was 16.1 ± 22 per hour. Hypertension (43.2%) and diabetes mellitus (37.3%) were the most common comorbidities: Mild OSA 28.9%, Moderate OSA 15.6%, and severe 16.4%. The severity of apnea increased with age and BMI classes. The prevalence of hypertension increased with the severity of apnea: 42.9% in mild, 47.4% in moderate, and 54.6% in severe AHI. The prevalence of coronary artery disease (CAD), congestive heart failure (CHF), and diabetes mellitus (DM) increased with the severity of apnea. Comorbidities was more among OSA patients with excessive sleepiness. After adjustment for age and gender, greater proportional odds of severe AHI were observed in males (aOR = 1.8), 30–59 years (aOR = 2.064), 60 years or above (aOR = 2.873), obese class II (aOR = 2.016), obese class III (aOR = 2.527), and in patients with hypertension (aOR = 1.272).

**Conclusion:**

Hypertension and obesity were highly prevalent in the study cohort and were associated with greater proportional odds of severe AHI.

## Background

Obstructive sleep apnea (OSA) is a serious health problem affecting not only an individual’s sleep but also health in general including cardiopulmonary, endocrine, vascular, and central nervous systems [1–7]. Nevertheless, OSA is not given much public attention in terms of screening and diagnosis. OSA is diagnosed with the use of polysomnography (PSG) testing by recording repetitive episodes of upper airway closure during sleep [8, 9]. OSA is established in terms of the Apnea–Hypopnea Index (AHI), which is the number of apnea–hypopnea/per hour measured during a sleep study [8–12]. AHI ≥ 5 per hour is used to diagnose OSA [8–10, 13, 14]. OSA severity is classified as follows: AHI < 5 per hour (normal), 5–15 AHI per hour (mild sleep apnea), ≥ 15 and < 30 AHI per hour (moderate sleep apnea), and AHI ≥ 30 per hour (severe sleep apnea) [9]. Untreated sleep apnea is associated with major comorbidities and health problems [1, 2, 15–17]. The prevalence of sleep apnea is considerably higher in patients with comorbidities than within the general population [5].

Few local studies revealed a high prevalence of OSA among the Saudi adult population [18–21]. Most of the studies conducted in Saudi Arabia used the Berlin Questionnaire (BQ) to screen for symptoms of sleep apnea. Based on the BQ, high-risk patients for sleep apnea were present in three out of ten of the population [18–20]. A major limitation of these studies is the use of subjective sleep assessments such as the Berlin Questionnaire rather than a formal sleep study or polysomnography. Regardless, sleep apnea in patients with comorbidities in Saudi Arabia is not well studied. We hypothesized that comorbidities such as diabetes, hypertension, and obesity increase with severe sleep apnea. This study aimed to determine whether there was a trend between the severity of sleep apnea and comorbidities controlling for demographic characteristics.

## Methods

A retrospective charts review was conducted at the Sleep Disorders Center (SDC) between 2003 and 2019. The SDC was established in 2003 at the King Abdulaziz Medical City in Riyadh (KAMC-R), Saudi Arabia. KAMC-R, with a capacity of 1800 beds, is considered as one of the biggest tertiary hospitals in Saudi Arabia and the Middle East. The study obtained ethical approval from the Ministry of National Guard—Health Affairs, Institution of Research Board (IRB) registered under number # RC15/058/R.

We have established a registry for all patients referred to our sleep disorders center since 2003. All patients filled out questionnaires concerning demographics, sleep symptoms, and comorbidities. All relevant data were collected, including anthropometric measurements (age, gender, body mass index (BMI), neck size, and Mallampati score), comorbidities, sleepiness scale as per the Epworth Sleepiness Scale (ESS) [22], sleep study results, and final diagnosis. All patients underwent standard in-lab type PSG type I (PSG). The PSG recording was performed using Alice^®^ 5 and Alice^®^ 6 diagnostic equipment (Respironics Inc., Murrysville, PA, USA). Manual scoring of the electronic raw data was performed by expert certified sleep technologists by following established criteria [23]. Hypopnea was defined as a reduction in airflow of ≥ 30% of the baseline that lasted for at least 10 s and resulted in either a ≥ 3% decrease in oxygen saturation from the pre-event baseline or arousal. Apnea was defined as a drop in the peak thermal sensor excursion greater than or equal to 90% of baseline for at least 10 s. The event was scored as obstructive apnea in the presence of continued respiratory effort. In this study, the study outcome, Apnea–Hypopnea Index (AHI), is categorized into four groups: normal (AHI < 5) per hour, mild (5 ≥ AHI < 15) per hour, moderate (15 ≥ AHI < 30) per hour, and severe (AHI ≥ 30) per hour [8, 9]. The Epworth Sleepiness Scale (ESS) was used to assess sleepiness in eight different situations on a scale of 0 (never doze) to 3 (high chance of dozing). The overall score is classified into four ordinal groups: normal 0–10, mild 11–14, moderate 15–17, and severe 18–24 [22, 24]. A cohort of 6030 patients who underwent PSGy during the study period was included in the analysis. We excluded all therapeutic sleep studies, narcolepsy, or less than 3 h of total sleeping time. We included 4391 patients in this analysis. All scored sleep studies were reviewed by one senior certified sleep technologist using AASM manual scoring of sleep and associated events [23].

The study population includes adults’ patients (18 years or above) who were suspected of sleep apnea and were referred to the in-lab polysomnographic sleep study. The study retrieved data on demographic characteristics such as age and gender. Data about patient clinical characteristics were collected (e.g., comorbidities such as hypertension, diabetes mellitus, coronary artery disease, obesity, congestive heart failure, and bronchial asthma).

### Data Analysis

SAS v 9.4 (SAS Institute Inc., Cary, NC, USA) was used for data analysis. Overall comorbidities and sample descriptive analyses were reported by frequency distributions. The frequency distributions of demographic and comorbidities variables were classified by the severe sleep apnea index and severity of sleepiness as per ESS and were tested by chi-squared methods. Since severity of apnea—normal, mild, moderate, and severe AHI—is ordinal, we performed the ordinal logistic regression model to assess the association between demographic and comorbidities variables and the likelihood of the severe sleep apnea index. We estimated adjusted odds ratio (aOR) and 95% confidence interval (95% CI). The assumption of proportional odds was assessed using a chi-squared score test. A *P* value greater than 0.05 indicates that the ordered logit coefficients are equal across the severity of the sleep apnea index.

## Results

We reported overall frequency distributions of demographic characteristics and comorbidities in Table 1. Gender was distributed equally in the study sample. The average age was 49.6 ± 14.8 years and the average AHI was 16.1 ± 22 per hour. Hypertension (43.2%) and diabetes mellitus (37.3%) were the most common comorbidities.

**Table 1:** Comorbidities and sample characteristics *n* = 4391

	*n*		%	
Gender				
Male	2224		50.8	
Female	2154		49.2	
Age				
29 years or less	428		9.7	
30–59 years	2813		64.1	
60 years or above	1150		26.2	
BMI				
Underweight	21		0.5	
Normal	178		4.2	
Overweight	592		13.9	
Obese class I	906		21.2	
Obese class II	804		18.9	
Obese class III	1764		41.4	
ESS ≥ 11				
Yes	1642		42.7	
No	2201		57.3	
Hypertension				
Yes	1893		43.2	
No	2489		56.8	
Coronary artery disease				
Yes	122		2.8	
No	4267		97.2	
Congestive heart failure				
Yes	175		4.0	
No	4215		96.0	
Bronchial asthma				
Yes	938		21.4	
No	3451		78.6	
Diabetes mellitus				
Yes	1637		37.3	
No	2749		62.7	

Table 2 compares demographics and comorbidities across different severity of apnea: normal, mild, moderate, and severe AHI. Of the sample, 1713 (39.1%) patients had normal AHI, 1269 (28.9%) patients had mild AHI, 687 (15.6%) patients had moderate AHI, and 722 (16.4) patients had severe AHI. There was an indicative difference in severity of apnea by gender (*P* = 0.001). Severe AHI (57.8%) was significantly more common among males, compared to 42% common among females (*P* = 0.001).

**Table 2:** Prevalence of comorbidities and its relation to severe sleep apnea

	Apnea hypopnea index								*P*
	None		Mild		Moderate		Severe		
	1713 (39.1%)		1269 (28.9%)		687 (15.6%)		722 (16.4%)		
	*n*	%	*n*	%	*n*	%	*n*	%	
Gender									
Male	803	47.0	660	52.1	346	50.6	415	57.8	0.001
Female	907	53.0	606	47.9	338	49.4	303	42.2	
Age									
29 years or less	249	14.5	111	8.7	42	6.1	26	3.6	0.001
30–59 years	1112	64.9	827	65.2	436	63.5	438	60.7	
60 years or above	352	20.5	331	26.1	209	30.4	258	35.7	
ESS ≥ 11									
Yes	627	41.1	460	41.7	245	41.3	310	49.9	0.001
No	899	58.9	643	58.3	348	58.7	311	50.1	
BMI									
Underweight	16	1.0	5	0.4	0	0.0	0	0.0	0.001
Normal	95	5.7	47	3.8	16	2.4	20	2.8	
Overweight (BMI 25.0–29.9)	265	15.9	174	14.2	89	13.3	64	9.1	
Obese class I (BMI 30.0–34.9)	366	21.9	272	22.2	133	19.9	135	19.2	
Obese class II (BMI 35.0–39.9)	300	18.0	211	17.2	137	20.5	156	22.2	
Obese class III) (BMI > 40)	627	37.6	516	42.1	294	43.9	327	46.6	
Hypertension									
Yes	631	36.9	543	42.9	325	47.4	394	54.6	0.001
No	1079	63.1	723	57.1	360	52.6	327	45.4	
Coronary artery disease									
Yes	32	1.9	31	2.4	28	4.1	31	4.3	0.001
No	1681	98.1	1236	97.6	659	95.9	691	95.7	
Congestive heart failure									
Yes	50	2.9	52	4.1	33	4.8	40	5.5	0.012
No	1662	97.1	1217	95.9	654	95.2	682	94.5	
Bronchial asthma									
Yes	383	22.4	257	20.3	142	20.7	156	21.6	0.550
No	1330	77.6	1010	79.7	545	79.3	566	78.4	
Diabetes mellitus									
Yes	553	32.3	462	36.4	295	43.0	327	45.4	0.001
No	1158	67.7	806	63.6	391	57.0	394	54.6	

*AHI* apnea-hypopnea index, *BMI* Body Mass Index, *ESS* Epworth Sleepiness Scale

The severity of apnea increases with age (*P* = 0.001) and BMI (*P* = 0.001). The prevalence of hypertension (*P* = 0.001) increases with the severity of apnea: 36.9% in patients with normal AHI, 42.9% in mild, 47.4% in moderate, and 54.6% in patients with severe AHI. The prevalence of coronary artery disease (*P* = 0.001), congestive heart failure (*P* = 0.012), and diabetes mellitus (*P* = 0.001) increase with the severe apnea. Sleepiness (ESS ≥ 11) was common in severe AHI (*P* = 0.001).

Table 3 illustrates the prevalence of comorbidities and its relation to the severity of OSA with EDS (ESS > 11) and without sleepiness. OSA with significant EDS was more common in male, obesity and older patients. The prevalence of hypertension, congestive heart failure, and diabetes mellitus were more significant among patient with OSA and EDS. EDS without OSA was more among young patients.

**Table 3:** Comorbidities and demographic characteristics of OSA severity with and without EDS

	Neither EDS nor OSA (control)		OSA without EDS (AHI ≥ 5 and ESS < 11)		EDS without OSA (ESS ≥ 11 and AHI < 5)		OSA and EDS ( AHI ≥ 5 and ESS ≥ 11)		*P*
	899 (20.47%)		1302 (29.65%)		627 (14.28%)		1015 (23.12%)		
	*n*	%	*n*	%	*n*	%	*n*	%	
Gender									
Male	404	20.8	656	33.8	310	16.0	571	29.4	< 0.001
Female	493	26.0	645	34.0	317	16.7	442	23.3	
Age									
29 years or less	133	35.6	94	25.1	83	22.2	64	17.1	< 0.001
30–59 years	600	24.0	860	34.4	407	16.3	636	25.4	
60 years or above	166	17.2	348	36.0	137	14.2	315	32.6	
BMI									
Underweight	8	50.0	5	31.3	3	18.8	0	0.0	< 0.001
Normal	57	36.5	43	27.6	29	18.6	27	17.3	
Overweight	140	27.1	182	35.3	91	17.6	103	20.0	
Obese class I	200	24.6	262	32.2	139	17.1	212	26.1	
Obese class II	145	20.1	220	30.5	130	18.0	226	31.3	
Obese class III	338	21.5	581	36.9	226	14.3	430	27.3	
HTN									
Yes	323	19.1	618	36.6	245	14.5	503	29.8	< 0.001
No	574	26.7	682	31.8	382	17.8	508	23.7	
CAD									
Yes	16	15.4	43	41.3	11	10.6	34	32.7	< 0.037
No	883	23.6	1259	33.7	616	16.5	979	26.2	
CHF									
Yes	20	13.7	61	41.8	17	11.6	48	32.9	< 0.004
No	878	23.8	1241	33.6	610	16.5	967	26.2	
BA									
Yes	197	23.5	279	33.2	150	17.9	214	25.5	< 0.565
No	702	23.4	1022	34.1	477	15.9	800	26.7	
DM									
Yes	277	18.9	514	35.2	221	15.1	450	30.8	< 0.001
No	620	26.1	786	33.1	406	17.1	564	23.7	

When controlling for the demographic and comorbidities in Table 4, the ordinal logistic regression suggested that male gender, age, BMI, and hypertension were independent risk factors of severe AHI. Unlike unadjusted analysis, sleepiness as per the ESS was not associated with severe AHI.

**Table 4:** Independent risk factors of severe sleep apnea

Parameter		Estimate	Standard error	Wald chi-square	*P*	95% CI for aOR		
						aOR	LCL	UCL
Intercept	1	− 2.002	0.156	165.393	0.001*			
Intercept	2	− 1.089	0.153	50.517	0.001*			
Intercept	3	0.158	0.152	1.069	0.301			
Gender	Male	0.294	0.034	73.761	0.001*	1.800	1.574	2.058
Age	30–59 years	0.131	0.047	7.768	0.005*	2.064	1.653	2.578
	60 years or above	0.462	0.061	56.884	0.001*	2.873	2.218	3.722
BMI	Underweight	− 0.733	0.471	2.427	0.119	0.652	0.208	2.047
	Overweight	− 0.067	0.121	0.303	0.582	1.269	0.895	1.798
	Obese class I	0.086	0.114	0.571	0.450	1.478	1.057	2.068
	Obese class II	0.396	0.116	11.723	0.001*	2.016	1.433	2.835
	Obese class III	0.622	0.109	32.455	0.001*	2.527	1.820	3.511
ESS ≥ 11	Yes	0.041	0.031	1.831	0.176	1.086	0.964	1.224
Hypertension	Yes	0.120	0.037	10.880	0.001*	1.272	1.103	1.467
Coronary artery disease	Yes	0.126	0.094	1.797	0.180	1.287	0.890	1.862
Congestive heart failure	Yes	0.084	0.080	1.104	0.293	1.182	0.865	1.616
Bronchial asthma	Yes	− 0.073	0.038	3.731	0.053	0.865	0.747	1.002
Diabetes mellitus	Yes	0.055	0.037	2.198	0.138	1.116	0.965	1.290

^*^Significant at *α* = 0.05

**Fig. 1:**
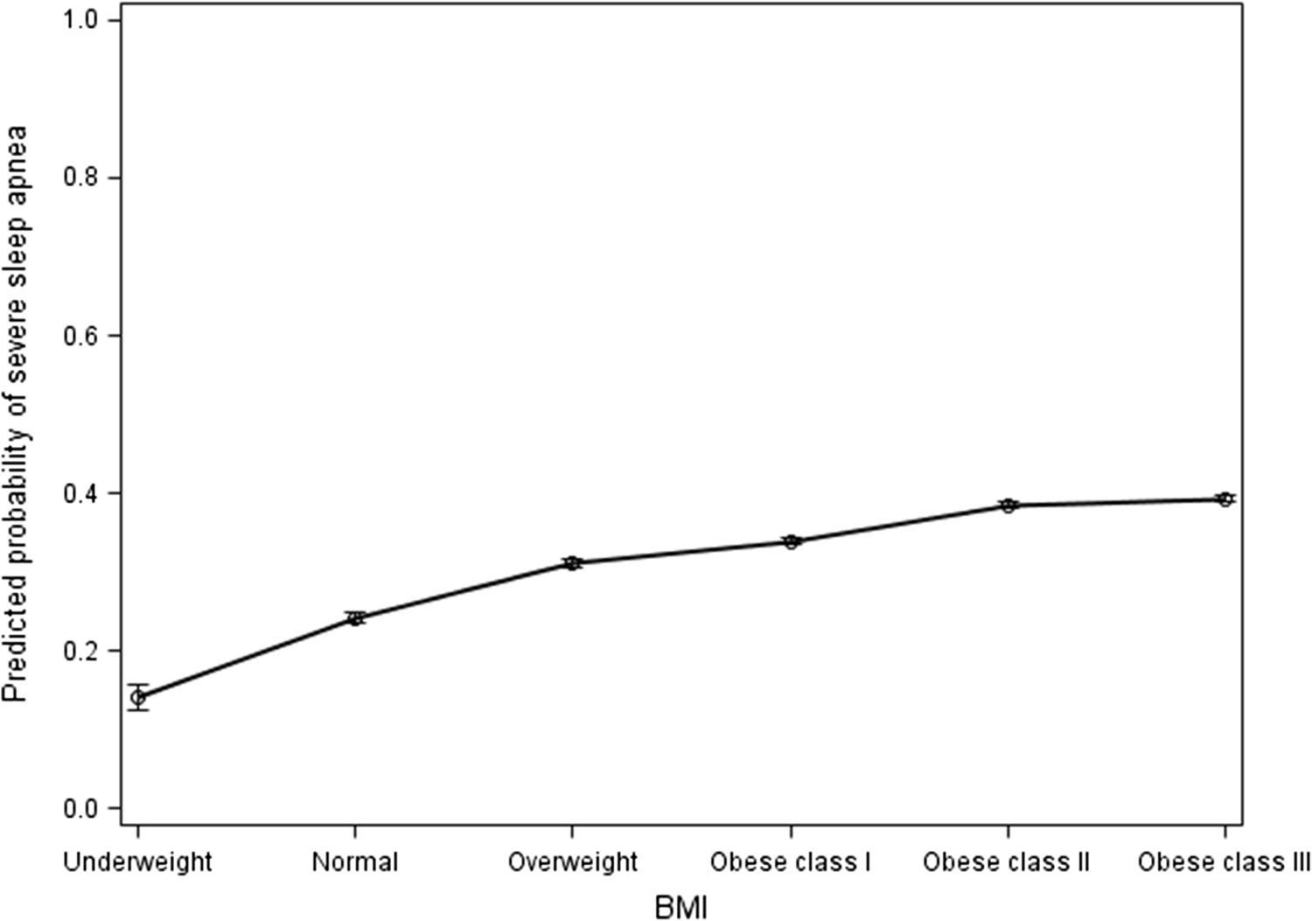
BMI classes in relation to severe apnea hypopnea index

The proportional odds of severe AHI were greater in males compared to females (aOR = 1.8). The severity of AHI was greater in 30–59 years (aOR = 2.064) and in 60 years or above (aOR = 2.873) compared to 29 years or less, Fig. 1. The severity of AHI was greater in obese class II (aOR = 2.016) and in obese class III (aOR = 2.527) compared to normal BMI, Fig. 1.

**Fig. 2:**
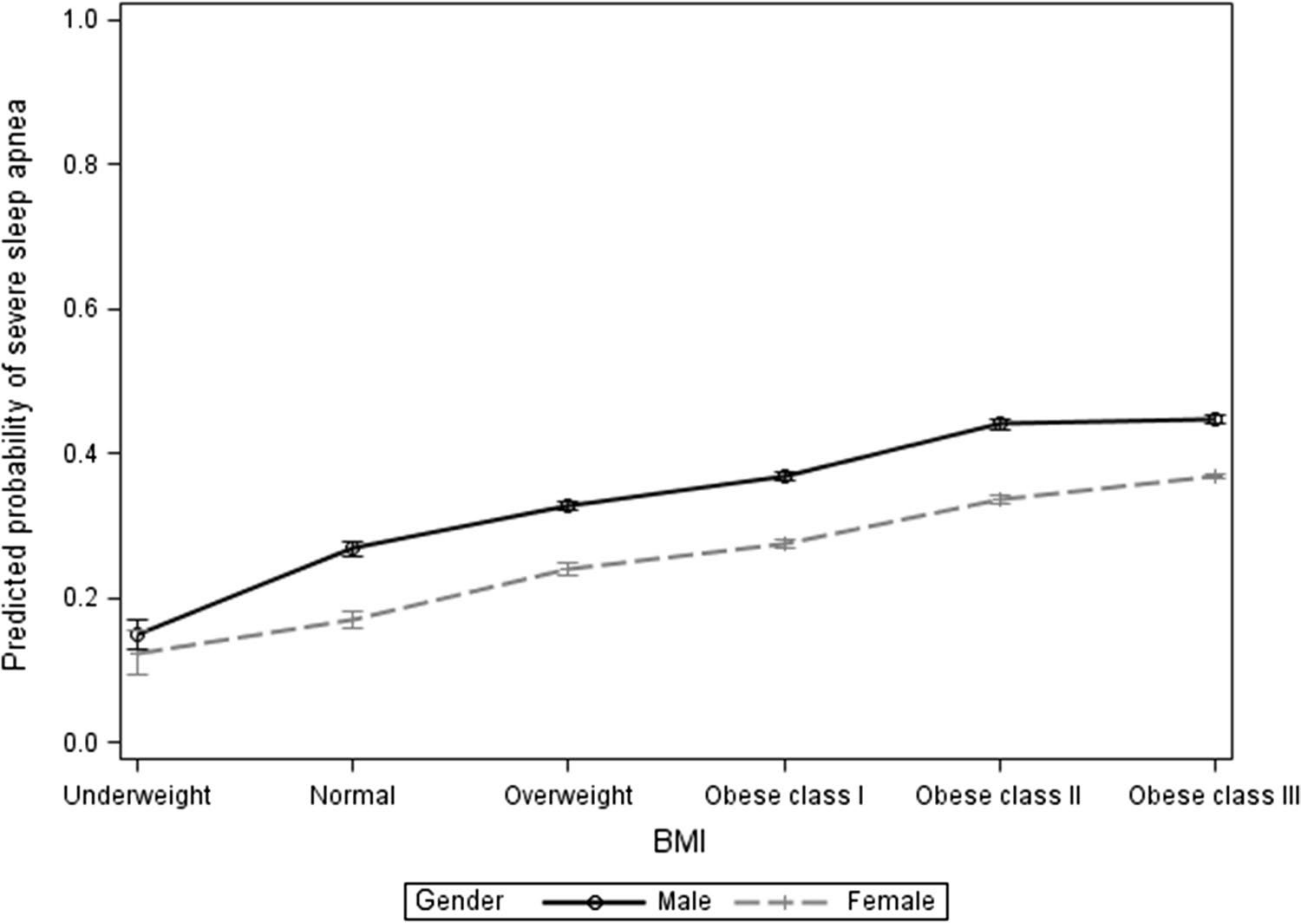
BMI classes in relation to severe apnea hypopnea index by gender

**Fig. 3:**
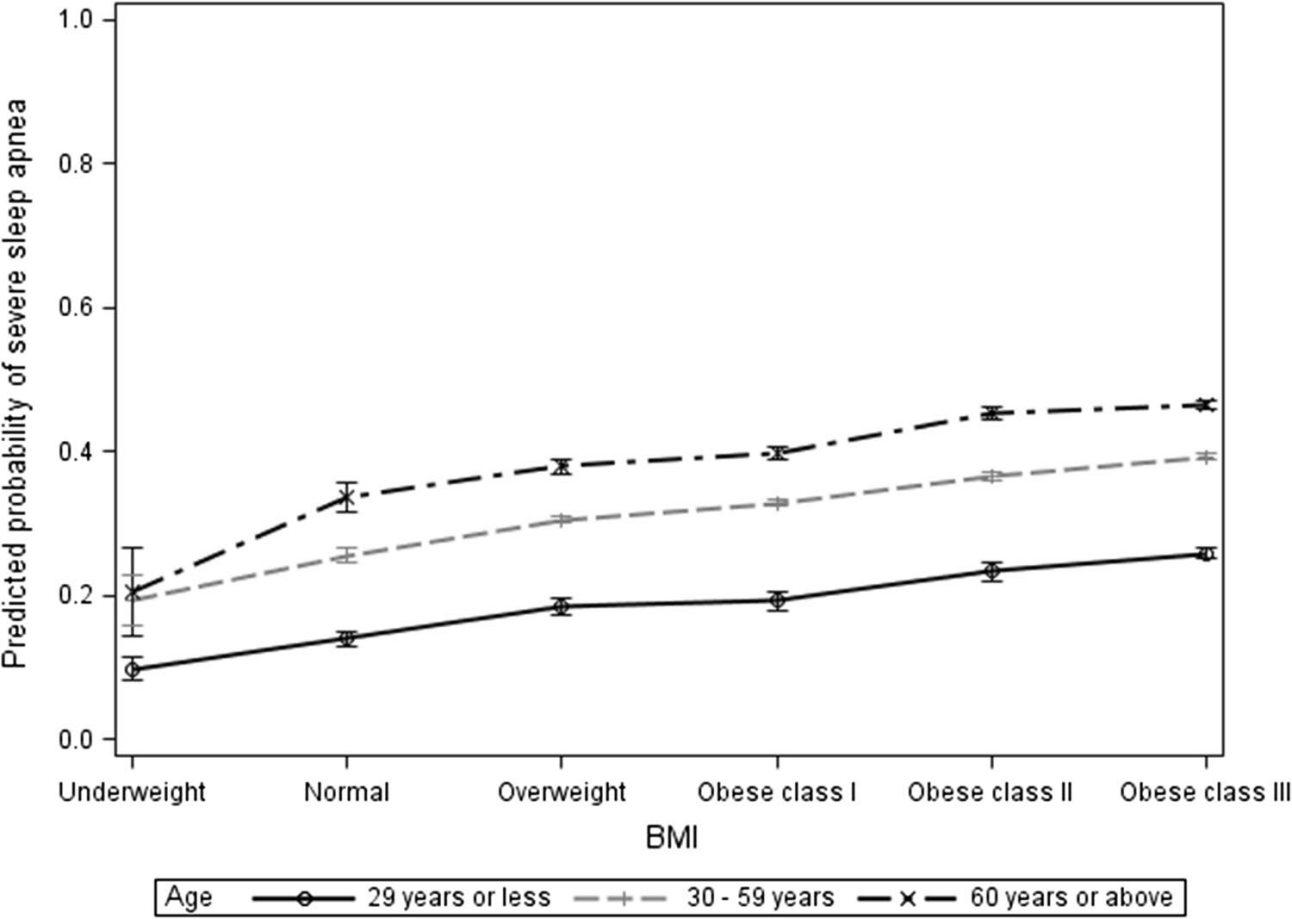
BMI classes in relation to severe apnea hypopnea index by age groups

Hypertension was associated with increased proportional odds of severe AHI (aOR = 1.272). Figures 2 and 3 illustrate BMI classes in relation to severe Apnea–Hypopnea Index by gender and age groups. The proportional odds assumption for this model was met, *P* value = 0.4540.

## Discussion

There is a shortage of knowledge about the prevalence of comorbidities in Saudi Arabia in contrast to other populations. OSA is highly prevalent in the general population and occurs in all age groups and more in female than male [18–20, 25]. In this study, OSA was more in male compared to female. The higher prevalence of OSA among male in this study is consistent with reports from other countries [26]. Furthermore, compared to other local studies, we used objective diagnostic sleep study, PSG while other prevalence studies used questionaries [18–20]. This study is not about the prevalence of OSA among general population but among cases refereed to sleep center to role out OSA. Therefore, this result cannot be generalized. The main strength of the present study is the large sample size, and the description of the largest national experience. Furthermore, all the data were collected at the time of the sleep study, included an equal number of genders, and all the study scoring was reviewed by one senior certified technologist, avoiding any bias in scoring. It provides information about the association between OSA and comorbidities. Similar to other studies, we reported a higher prevalence of comorbidities among OSA [29, 30] and a higher prevalence of comorbidities in severe OSA and EDS [5, 27]. EDS is frequently reported by patients with OSA but is not invariably present. The overall prevalence of EDS in our population was 43%, and significantly high in severe OSA. The relation between OSA and EDS remains controversial [28–30]. EDS in OSA may indicate more arousals and is associated with a higher risk of comorbidities [7, 31, 32]. Nevertheless, recent studies reported the presence of EDS in sleep apnea patients associated with increases the risk of DM, obesity, hypertension, heart failure, and coronary heart disease [6, 7, 29–31, 33–35]. The present study EDS in OSA was associated with a higher risk of comorbidities. A higher prevalence of comorbidities including hypertension among our male patients is probably due to a higher prevalence of severe OSA among males compared to females (OR = 1.89). The Alharbi et al. study about the prevalence of hypertension among OSA patients reported a higher prevalence of hypertension among female patients [22]. However, this study includes a much larger number of participants, and this may reflect a true higher prevalence of hypertension among healthy male Saudis [33]. The prevalence of OSA is strongly associated with overweight and obesity [36, 37]. Obesity is quite prevalent among Saudi population. An epidemiological study about obesity among Saudis including 10,735 participants found 28.7% were obese (BMI ≥ 30 kg/m), which was higher among women (33.5% vs. 24.1%) [38]. The present study, obesity prevalence was higher among OSA patients (88%) and, using ordinal logistic regression obesity was an independent factor for OSA. The proportional odds of severe AHI were greater in obese classes II and III compared to normal BMI. Normal sleep study reported in almost more than third of our study sample, this is because we only recently used home sleep studies and in our center, we receive frequent referrals preoperative consultation to role out OSA before bariatric, ENT, and maxillofacial surgeries and to role sleep apnea as a cause of pulmonary hypertension and EDS.

The association of bronchial asthma and OSA is controversial. A systematic review by Davies et al. [44] found a weak association between asthma and OSA. The asthma prevalence in our study was 21.95% but we did not find any association between asthma and OSA or asthma.

Limitations include a retrospective design; therefore, no cause-and-effect relationship could be determined. It is a single-center experience, and it did not report the effect of the treatment on the severity or outcome. We encourage all sleep centers in the country to adopt registry information about OSA, to describe the prevalence, comorbidities, epidemiological patterns, and outcomes of OSA.

## Conclusion

Our study revealed a high prevalence of associated comorbidities in patients with OSA and/or EDS.

## Abbreviations

OSA: Obstructive sleep apnea
AHI: Apnea–Hypopnea Index
CAD: Coronary artery disease
CHF: Congestive heart failure
BQ: Berlin Questionnaire
ESS: Epworth Sleepiness Scale
SDC: Sleep Disorders Center
PSG: Polysomnography I
95% CI: Confidence interval
IRB: Institution of Research Board
DM: Diabetes mellitus
